# Identification of Body Size Determination Related Candidate Genes in Domestic Pig Using Genome-Wide Selection Signal Analysis

**DOI:** 10.3390/ani12141839

**Published:** 2022-07-19

**Authors:** Bing Pan, Haoyuan Long, Ying Yuan, Haoyuan Zhang, Yangyang Peng, Dongke Zhou, Chengli Liu, Baiju Xiang, Yongfu Huang, Yongju Zhao, Zhongquan Zhao, Guangxin E

**Affiliations:** 1 College of Animal Science and Technology, Southwest University, Chongqing 400716, China; zyjjjzpdyx@163.com (B.P.); asrimoom@gmail.com (H.L.); 15703001951@163.com (Y.Y.); swuzhanghy@163.com (H.Z.); seraphicluv@icloud.com (Y.P.); zhoudongke@sjtu.edu.cn (D.Z.); lcl222333@outlook.com (C.L.); h67738337@swu.edu.cn (Y.H.); zyongju@163.com (Y.Z.); zhongquanzhao@126.com (Z.Z.); 2 Chongqing Academy of Animal Sciences, Chongqing 402460, China; ja_9700@126.com

**Keywords:** domestic pigs, body size, genome wide, skeletal muscles

## Abstract

**Simple Summary:**

In this study, the pairwise fixation index (F_ST_) and π ratio (case/control) genetic parameters were used to identify the genes that influence the body size of pigs and analyze the genetic basis of pig body size formation. The results of candidate gene (CG) annotation showed that a series of CGs (*MSTN*, *LTBP4*, *PDPK1*, *PKMYT1*, *ASS1*, and *STAT6*) was enriched into the gene ontology terms. Moreover, molecular pathways, such as the PI3K-Akt, HIF-1, and AMPK signaling pathways, were verified to be related to body development. These findings will help us further understand the genetic basis of animal body-size determination.

**Abstract:**

This study aimed to identify the genes related to the body size of pigs by conducting genome-wide selection analysis (GWSA). We performed a GWSA scan on 50 pigs belonging to four small-bodied pig populations (Diannan small-eared pig, Bama Xiang pig, Wuzhishan pig, and Jeju black pig from South Korea) and 124 large-bodied pigs. We used the genetic parameters of the pairwise fixation index (F_ST_) and π ratio (case/control) to screen candidate genome regions and genes related to body size. The results revealed 47,339,509 high-quality SNPs obtained from 174 individuals, while 280 interacting candidate regions were obtained from the top 1% signal windows of both parameters, along with 187 genes (e.g., *ADCK4*, *AMDHD2*, *ASPN*, *ASS1*, and *ATP6V0C*). The results of the candidate gene (CG) annotation showed that a series of CGs (e.g., *MSTN*, *LTBP4*, *PDPK1*, *PKMYT1*, *ASS1*, and *STAT6*) was enriched into the gene ontology terms. Moreover, molecular pathways, such as the PI3K-Akt, HIF-1, and AMPK signaling pathways, were verified to be related to body development. Overall, we identified a series of key genes that may be closely related to the body size of pigs, further elucidating the heredity basis of body shape determination in pigs and providing a theoretical reference for molecular breeding.

## 1. Introduction

As one of the earliest domesticated animals, pigs are affected by different long-term artificial selection processes and the natural environment of their habitats. Consequently, pig breeds exhibit numerous evident differences in phenotype appearance and growth performance, particularly in terms of body size [1,2,3]. Some breeds, such as long white, Large White, and Duroc pigs, are larger, with adults weighing more than 200 kg. Other breeds, such as Bama Xiang and Wuzhishan pigs, are smaller, with adults generally weighing below 50 kg [4].

Breeds with smaller weight and size are frequently referred to as miniature pigs. Miniature pigs are extensively used in many fields apart from animal husbandry. For example, they have become ideal laboratory animals because of their small size, low feed consumption, genetic stability, easy microbial control, convenient operation and management, and docile temperament [5,6]. In particular, given that the physiological and anatomical characteristics of pigs are highly similar to those of humans, these animals can be used as important medical models [7,8,9]. They can also be bred as pets because of their advantages in terms of appearance [10]. Research on miniature pig breeds has become increasingly comprehensive, and the use of these pigs has become more common. For example, miniature pigs are selected as medical models for respiratory toxicology [11,12], reproductive toxicity [13], atherosclerosis [14,15], diabetes [16,17], and neurodegenerative diseases [18,19]. At present, a large number of genes related to body size development have been verified; they include *HMGA2*, *BMP2*, *FGFR3*, and insulin-like growth factor (IGF)-1R.

In particular, studies have confirmed that the HMGA2-mediated JNK signaling pathway can affect the differentiation of osteoblasts. Evidence of a close link between the expression of the *HMGA2* gene and pig body size has also been found; that is, the *HMG*A2 gene is only activated during infant development and it controls the total number of cells in an animal; in particular, its expression level is proportional to animal body size [20,21]. Simultaneously, previous studies have proven that the *BMP2* and *FGFR3* genes play key roles in the cartilage and bone formation of pigs. The *BMP2* gene promotes pig bone development; conversely, the *FGFR3* gene inhibits bone development in pigs [22]. Furthermore, evidence has revealed that mice with *IGF1R* deficiency will suffer from dwarfism to a certain extent, and *IGF1R* defects can alter chondrocyte proliferation, leading to the excessive hypertrophy of growth plates in the bone extension zone and apoptosis [21].

Although studies that used candidate gene (CG) markers to explore the genetic basis of body shape in miniature pigs are extensive [23,24,25], these markers are not yet fully understood at present. Accordingly, the current study aimed to compare the genetic divergence between miniature and large pigs worldwide to recognize the genetic mechanism behind miniature pigs with published SNP data from swine genome sequencing data. To achieve this objective, we screened highly selective regions in miniature pigs to identify CGs that may affect their body size compared with large pigs. The results could provide a theoretical reference for the experimental model breeding of miniature pigs.

## 2. Materials and Methods

A public dataset that contained 174 pig genome [24,26,27,28,29,30,31,32] raw SNPs mapped by the pig reference genome [Sscrofa10.2 (GCA_000003025.4); Table S1; http://ftp.ensembl.org/pub/release-89/fasta/sus_scrofa/dna/ (accessed on 3 July 2021)] was obtained from the Genome Variation Map (https://ngdc.cncb.ac.cn/gvm/ (accessed on 13 February 2022)). It included data from 50 small-bodied (case group) and 124 large-bodied (control group) pigs, as detailed in Table S1. Moreover, the obtained genetic risk score data were subjected to quality control detection by using fastp (v0.20.1). SNP sites with an average minimum allele frequency of ≤0.05 were removed, and a high-quality dataset with 47,339,509 SNPs was obtained.

In the current study, the genome-wide selection analysis strategy was performed with the pairwise fixation index (F_ST_) [33] and π ratio (case/control) [34] by using 40 kb-long windows and 10 kb step size with VCFtools (http://vcftools.sourceforge.net/ (accessed on 25 February 2022)) [35].

The interacted windows of both parameters with the top 1% windows were obtained, and overlapped genes were annotated with the variant effect predictor. In addition, CGs were subjected to gene ontology (GO) and the Kyoto Encyclopedia of Genes and Genomes (KEGG) by using KOBAS 3.0 (http://kobas.cbi.pku.edu.cn/; accessed on 16 February 2022). The significant enrichment thresholds were defined as *p* ≤ 0.05. The formula for calculating the *p* value of KEGG and GO enrichment was as follows:

(1) $$Ρ=1-\sum_{i=0}^{m-1}\frac{\left(\begin{array}{c} M\\ i \end{array}\right)\left(\begin{array}{c} N-M\\ n-i \end{array}\right)}{\left(\begin{array}{c} N\\ n \end{array}\right)}$$

where *N* is the number of all genes with GO annotations, n is the number of genes in *N*, *M* is the number of all genes that are annotated to the specific GO terms, and *m* is the number of genes in *M*.

## 3. Results

**Figure 1:**
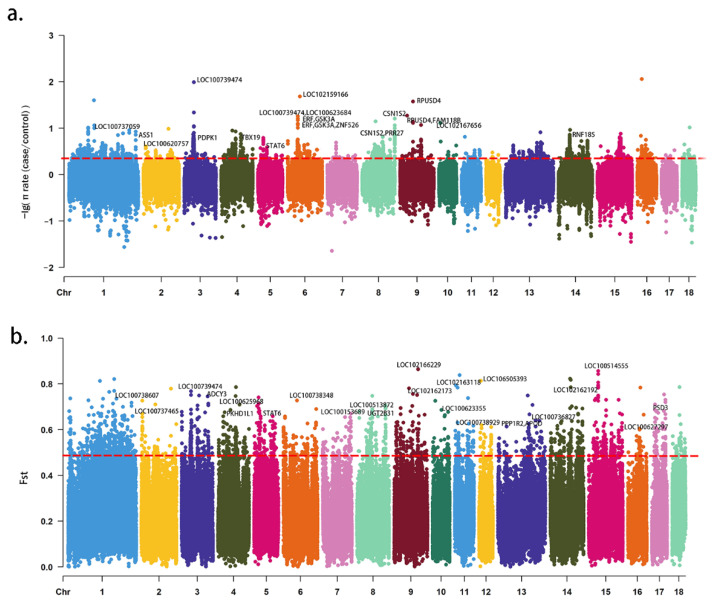
Wide-genome selective signal analysis of 174 pigs by SNP dataset. (**a**) The distribution of θπ ratio (−Log10[θπ ratio(θπcase/θπcontrol)]) on the autosomal chromosome calculated by 40k sliding window size with 20k step. (**b**) The distribution of F_ST_ on the autosomal chromosomes calculated by 40k sliding window size with 20k step.

From the 174 pig genome-wide SNP data, 235,623 autosomal windows were obtained. The thresholds of the top 1% selective signal windows were defined as 0.48 (F_ST_) and 0.31 (π ratio) (Figure 1). A total of 187 CGs were identified from 280 interacted windows (Table S2).

**Figure 2:**
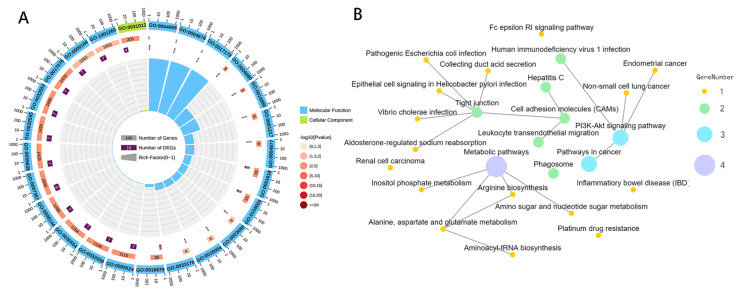
Annotation and functional enrichment of candidate genes of pig body size determination. Note: (**A**) Circle graphic of gene ontology (GO) enrichment. (**B**) Network graphic pattern of top 25 significant enriched molecular signaling pathways (KEGG).

The GO analysis results revealed that 40 CGs were enriched to 369 GO terms. Among the 369 enriched GO terms, 210 were biological processes. In particular, cell adhesion was the most significant (including a variety of signaling pathways that regulate cell physiological processes), 76 were cellular components, and 76 were molecular functions. In particular, 189 enriched terms were significant (*p* < 0.05) (Table S3, Figure 2A).

In accordance with the functional classification of a GO term, a series of CGs was enriched into GO terms related to muscle development regulation, such as cell growth regulation (*LTBP4*) and skeletal muscle atrophy (*MSTN*). In addition, it enriches galvanic processes related to cell appreciation, such as growth hormone secretion (*LTBP4*), mitotic cell cycle (*TUBB3* and *PKMYT1*), growth hormone receptor signaling pathway via JAK-STAT (*STAT6*), growth hormone response (*ASS1*), mitotic nuclear division regulation (*PKMYT1*), insulin receptor binding (*PDPK1*), and cell growth regulation (*LTBP4*). In particular, several GO terms related to bone growth and development were identified, such as the negative regulation of ossification (*KREMEN2*) and regulation of bone mineralization (*OMD*).

The results of KEGG enrichment showed that 20 genes were enriched to 88 KEGG signaling pathways (Table S4, Figure 2B). Among these enriched pathways, 26 belonged to the organizational system category, 7 belong to metabolism, 27 belong to human diseases, 3 belong to genetic information processing, 13 belong to environmental information processing, and 10 belong to cellular processes. A total of 12 enriched pathways were significant (*p* < 0.05), such as the PI3K-Akt signaling pathway, collecting duct acid secretion, and arginine biosynthesis.

Notably, 7 of the 20 CGs (e.g., *PDPK1*, *STAT6*, and *ASS1*) were not only enriched into metabolic-related signaling pathways, including the thyroid hormone signaling pathway, and the alanine, aspartate, and glutamate metabolism, but also enriched into known cell value-added growth regulation-related signaling pathways (e.g., cell cycle, apoptosis, JAK-STAT signaling pathways) and growth/development-related signaling pathways (e.g., phosphatidylinositol signaling system and HIF-1 signaling pathways).

## 4. Discussion

The growth of an animal’s body is accompanied by the proliferation and differentiation of various cells and the regulation of various types of growth hormones. In the current study, the growth and development of muscles, fats, and bones are important influencing factors. In this work, a series of high-selection signal regions and coding genes (e.g., *MSTN*, *LTBP4*, and *PDPK1*) related to body size was identified.

Evidence supports that cell number is the primary factor that affects body size in mammals. [36]. Cell proliferation and apoptosis are inextricably linked to the number of cells [37]. Studies have shown that systemic factors (e.g., growth hormone) and local signaling molecules (e.g., *IGF*) control the proliferation and apoptosis of various cells in the body, ultimately controlling body size [23,38].

For many types of cells, hypoxia induces decreased cell proliferation, because increasing cell numbers only exacerbates hypoxic stress, particularly in embryonic stem cells (ESCs) [39,40,41]. A previous study found decreased bromodeoxyuridine incorporation into DNA (a measure of DNA replication) when ESCs were exposed to hypoxia in mice. Conversely, ES and fibroblasts from mice with HIF-1α functional loss with exposure to hypoxia did not result in reduced DNA replication [40,42]. HIF-1α overexpression also reportedly induced cell cycle arrest [43].

The size of muscle tissues is directly related to body size [44]. Studies have shown that when the rate of protein anabolism exceeds that of protein catabolism, the result can be skeletal muscle growth [45]. Two well-known molecular signaling pathways are responsible for protein synthesis, namely, the IGF1-Akt-mTOR and inhibitin-Smad2/3 pathways, which positively and negatively regulate muscle growth, respectively [46]. The CG *MSTN* identified in this study was enriched in the aforementioned pathways. Numerous studies have indicated that the functional loosening of *MSTN* is primarily the genetic basis for the double muscle glute phenotype of robust muscle development in domestic animals [47]. *MSTN* has also been demonstrated to impair satellite cell activation, proliferation, and macrophage and myoblast migration to damage sites to inhibit skeletal muscle regeneration [48].

Moreover, the *LTBP4* gene identified in this study was confirmed to be associated with the clinical manifestations of muscular dystrophy in mice and humans [49]. For example, increasing the expression of the *LTBP4* gene significantly increased body weight and skeletal muscle mass in malnourished mice [50]. In particular, the increased expression level of *LTBP4* reduced *MSTN* expression [51]. LTBP4 has also been suggested to interact with GDF11 protein, which is highly associated with muscle growth inhibition, to codirect muscle development and regulation [52,53].

In addition, CG *PDPK1* was enriched into the AMPK signaling pathway (5′-adenosine monophosphate-activated protein kinase); this phenomenon has been extensively demonstrated to regulate cell anabolism and catabolism by modulating many downstream targets participating in skeletal muscle development and growth [54]. Pharmacological evidence shows that AMPK inhibits muscle growth, and AMPK activation inhibits protein translation increase after resistance exercise [55]. In theory, protein catabolism exceeding anabolism leads to skeletal muscle atrophy [51,56]. The activation of AMPK can reportedly exert the inhibitory effects of protein synthesis on multiple cell types [57,58], particularly muscle cells and cardiomyocytes [52,59].

Moreover, AMPK exhibits protein synthesis inhibition through the rapamycin complex 1 (mTORC1) pathway [60,61], and mTOR has also been verified as a critical regulating factor for skeletal muscle quality [62]. In particular, mTORC1 drives cell growth by stimulating downstream protein synthesis through phosphorylation, such as ribosomal protein S6 kinase (p70S6K1) and eukaryotic initiation factor 4E-binding protein 1 (4E-BP1) [63]. Studies have shown that mTOR can also be involved in regulating mitochondrial function. For example, mTOR can coordinate energy consumption and mitochondrial energy production during messenger RNA translation by stimulating the synthesis of mammalian nucleus-encoded mitochondrion-associated proteins (e.g., TFAM and mitochondrial ribosomal proteins) [64,65].

AMPK also performs catabolic regulation depending on the AMPK stimulation of *FoxO*. Several pieces of evidence have supported the theory that an injection of AMPK activator in mice increased the expression of *FoxO1* and *FoxO3* [66,67]. In skeletal muscles, two E3 enzymes (atrogin-1 and MuRF-1) have been confirmed to guide the polyubiquitination of proteins; these enzymes are also associated with muscle atrophy [68]. The expression of muscle atrophy-related genes, i.e., atrogin-1 and MuRF-1, is known to be regulated by transcription factors, particularly *FoxO* members [69]. Coincidentally, *FoxO6* has been shown to be involved in the dietary obesity and type 2 diabetes of animals via insulin resistance [70]. Therefore, the CG *PDPK1*, which was identified in the current study to be enriched in the mTOR, AMPK, FoxO, and other related pathways, may be an important genetic basis for determining the body size of pigs.

The region of the *STAT6* gene, which is involved in growth hormone (GH) secretion, was identified to be a highly selective signal in the current study. GH is a known pleiotropic hormone that coordinates extensive physiological processes, including growing effects on bones, muscles, and fats [71]. In particular, GH promotes anabolic effects in most tissues [72].

Nitric oxide (NO) has been verified to play a key role in regulating systemic metabolism and insulin sensitivity [73]. NO regulates aerobic respiration processes in the mitochondrion through mitochondrial activity and O_2_ levels [11,12]. The *ASS1* identified in the present study is the enzyme responsible for the metabolism of citrulline in mammals; meanwhile, the arginine succinate produced by *ASS1* is a direct precursor of arginine, which is the raw material for the most common route to the intracellular synthesis of NO [74].

The PI3K/AKT/mTOR pathway enriched by a large number of CGs in the current study has also been confirmed to be involved extensively in the growth and metabolism of cells and their maintenance [75]. Furthermore, bone mineral density and bone volume fraction in animals are known to be related to body size [76]. Recent studies have shown that the PI3K/Akt signaling pathway also collaborates with glucocorticoids to control osteoblast growth and differentiation by inhibiting osteoblast replication and function and promoting osteoblast apoptosis [77].

Finally, the CG *ITPKC* was identified in the current study to be enriched in the phosphatidyl inositol (PI) signaling pathway. Previous findings have demonstrated that *ITPKC* controls the biological functions of organelles by regulating vesicle transport and regulates lipid distribution and metabolism via lipid transfer proteins [78]. In particular, numerous clinical studies have shown that PI metabolism disorders are major causes of obesity and diabetes [79].

## 5. Conclusions

In the current study, a series of hormone secretion regulation, resting oxygen consumption of cell pathways, and related CGs was identified via whole genome resequencing technology to help understand the genetic basis of pig body-size determination.

## Data Availability

The data presented in this study are available in this article (and Supplementary Materials).

## Supplementary Materials

The following are available online at https://www.mdpi.com/article/10.3390/ani12141839/s1. Table S1: Information of all public 174 pig individuals’ genome dateaet; Table S2: Wide-genome selective single ananlysis and 187 candidate genes from interacted top 1% windows of FST and π rate; Table S3: Gene Ontology (CO) analysis of 187 candidate genes; Table S4: Kyoto Encyclopedia of Genes and Genomes (KEGG) analysis of 187 candidate genes.
